# Metabolic: week 48 comparison of metabolic parameters and biomarkers in subjects receiving darunavir/ritonavir or atazanavir/ritonavir

**DOI:** 10.1186/1758-2652-13-S4-P74

**Published:** 2010-11-08

**Authors:** T Overton, JA Aberg, S Gupta, R Ryan, B Baugh, G De La Rosa

**Affiliations:** 1 Washington University School of Medicine, St. Louis, USA; 2 NYU School of Medicine, New York, USA; 3 Indiana University School of Medicine, Indianapolis, USA; 4 Tibotec Inc, Titusville, USA; 5 Tibotec Therapeutics, Titusville, USA

## Purpose

Protease inhibitors may contribute to metabolic complications and cardiovascular risk associated with HIV infection. Here we investigate metabolic changes following treatment with darunavir/ritonavir (DRV/r)- compared with atazanavir/ritonavir (ATV/r)-based therapy.

## Methods

In this 48-week, Phase IV, multicenter, open-label, randomized, exploratory study, HIV-1—infected, antiretroviral naïve adults were given DRV/r 800/100mg once daily (qd) or ATV/r 300/100mg qd, both with tenofovir/emtricitabine 300/200mg qd. Week 48 changes in fasting lipids, glucose, insulin, insulin sensitivity, creatinine clearance, biomarkers (inflammation, coagulation and bacterial translocation), CD4 count, viral load (VL) and safety are reported. Observed values and descriptive statistics are reported through Week 48 for intent-to-treat and lipid evaluable populations.

## Results

34 (median age, 37 years; men, n=29) and 31 (median age, 35 years; men, n=27) subjects were randomized to the DRV/r arm and ATV/r arm, respectively. Of these, 29 DRV/r and 25 ATV/r subjects completed Week 48. At Week 48, changes in fasting lipids and biomarkers were similar between arms. Although rates of adverse events (AEs) and laboratory abnormalities were comparable between arms, ATV/r arm had higher rates of grade 2—4 hyperbilirubinemia (n=27 [87%] vs n=1 [3%]). At Week 48, 77% of DRV/r and 71% of ATV/r subjects had VL <50 copies/mL (confirmed virologic response). Table 1.

**Table 1:** 

Changes in metabolic and efficacy parameters from baseline to Week 48					
	**DRV/r**		**ATV/r**		**Difference in mean change between arms, (95% CI)**

	**BL**	**Change from BL at Week 48**	**BL**	**Change from BL at Week 48**	

**Fasting lipid parameters**^a^					

TG, mg/dL					

Mean (SD)	114 (57)	26 (69)	114 (84)	10 (74)	16.5 (—25.0, 58.0)

Median (IQR)	87 (77, 153)	11 (—15, 37)	90 (65, 119)	15 (—24, 54)	

TC, mg/dL					

Mean (SD)	142 (28)	22 (31)	165 (30)	12 (32)	10.5 (—7.7, 28.8)

Median (IQR)	136 (122, 159)	22 (10, 40)	163 (140, 183)	13 (—11, 29)	

LDL, mg/dL					

Mean (SD)	85 (22)	15 (26)	100 (24)	14 (27)	0.8 (—14.6, 16.3)

Median (IQR)	81 (71, 101)	17 (1, 30)	104 (79, 117)	8 (—7, 33)	

HDL, mg/dL					

Mean (SD)	38 (13)	6 (7)	45 (14)	4 (10)	2.3 (—2.8, 7.3)

Median (IQR)	37 (28, 44)	6 (2, 10)	44 (39, 49)	2 (—2, 11)	

TC/HDL ratio					

Mean (SD)	4.1 (1.14)	0.1 (1.06)	3.9 (1.02)	—0.1 (0.75)	0.20 (—0.34, 0.75)

Median (IQR)	4.08 (3.41, 4.90)	0.05 (—0.62, 0.67)	3.87 (3.10, 4.24)	—0.08 (—0.54, 0.26)	

Apo A1, mg/dL					

Mean (SD)	115 (26)	12 (16)	128 (22)	3 (19)	9.7 (—0.5, 19.8)

Median (IQR)	112 (96, 127)	11 (—1, 27)	127 (112, 132)	3 (—11, 19)	

Apo B, mg/dL					

Mean (SD)	74.5 (19)	4 (21)	81.7 (18.5)	2 (17)	2.0 (—9.3, 13.4)

Median (IQR)	72 (64, 84)	3 (—3, 15)	81 (66, 95)	4 (—10, 12)	

Apo B/A1 ratio					

Mean (SD)	0.68 (0.20)	0.68 (0.25)	0.65 (0.16)	0.66 (0.17)	—0.02 (—0.125, 0.079)

Median (IQR)	0.67 (0.55, 0.82)	0.63 (0.51, 0.79)	0.64 (0.56, 0.73)	0.68 (0.54, 0.78)	

**Fasting glucose, fasting insulin and HOMA-IR,^b^ mean (SD)**					

Glucose, mg/dL	89 (12)	3 (9)	90 (11)	6 (22)	—3.6 (—12.8, 5.6)

Insulin, uIU/mL	6 (6)	1 (6)	9 (14)	—3 (17)	3.8 (—3.0, 10.6)

HOMA-IR	1.62 (1.70)	0.04 (2.26)	2.94 (6.02)	—1.24 (8.01)	1.27 (—2.66, 5.20)

**Creatinine clearance,^b^ mean (SD)**					

Creatinine clearance, mL/min	107.6 (28.7)	—0.00 (0.288)	110.9 (27.9)	—0.03 (0.244)	0.03 (—0.12, 0.18)

**Biomarkers,^b^ mean (SD)**					

IL-1 Beta, pg/mL	0.2 (0.3)	0.3 (1.4)	0.3 (0.3)	—0.1 (0.3)	0.40 (—0.22, 1.04)

IL-6, pg/mL	1.9 (1.9)	0.2 (7.3)	1.0 (1.3)	0.3 (0.9)	—0.08 (—3.24, 3.08)

hs-CRP, mg/L	3.1 (5.2)	1.2 (11.2)	2.2 (2.5)	0.6 (5.1)	0.65 (—4.55, 5.85)

TNF-alpha, pg/mL	4207 (1702)	—1384 (1722)	2957 (727)	—442 (722)	—942.1 (—1735.3, —149.0)

LPS, pg/mL	85 (29)	—18 (35)	87 (31)	—17 (51)	—1.4 (—25.6, 22.9)

D-dimer, ng/mL	373 (580)	—192 (587)	189 (111)	—24 (144)	—168.0 (—432.2, 96.2)

Fibrinogen, g/L	3.3 (1.1)	—0.3 (1.1)	3.2 (0.7)	—0.3 (0.9)	0.02 (—0.60, 0.61)

**Efficacy parameters,^b^ mean (SD)**					

Viral load, log_10_ copies/mL	5.0 (0.8)	—3.3 (0.8)	4.6 (0.7)	—2.9 (0.7)	—0.4 (—0.8, 0.1)

CD4+ cell count, cells/mm^3^	268.3 (144.2)	217.4 (116.8)	326.7 (174.1)	205.3 (153.5)	12.1 (—61.8, 86.1)

^a^Lipid evaluable set; ^b^ITT-observed (sample size varies by time point and parameter);

DRV/r, darunavir/ritonavir; ATV/r, atazanavir/ritonavir; BL, baseline; CI, confidence interval; TG, triglycerides;

SD, standard deviation; IQR, interquartile range; TC, total cholesterol; LDL, low-density lipoprotein;

HDL, high-density lipoprotein; Apo, apolipoprotein; HOMA-IR, homeostasis model assessment-estimated insulin resistance;

IL, interleukin; hs-CRP, high-sensitivity c-reactive protein; TNF, tumor necrosis factor; LPS, lipopolysaccharide;

ITT, intent-to-treat

## Conclusions

Changes from baseline to Week 48 in fasting lipids, glucose, insulin, HOMA-IR, creatinine clearance, biomarkers, CD4 and VL were similar for DRV/r and ATV/r. Given its favorable metabolic profile, which was maintained over 48 weeks, DRV/r provides a valuable therapeutic option for HIV-1—infected subjects.

